# Aging and serum exomiR content in women-effects of estrogenic hormone replacement therapy

**DOI:** 10.1038/srep42702

**Published:** 2017-02-14

**Authors:** Reeta Kangas, Timo Törmäkangas, Vidal Fey, Juha Pursiheimo, Ilkka Miinalainen, Markku Alen, Jaakko Kaprio, Sarianna Sipilä, Anna-Marja Säämänen, Vuokko Kovanen, Eija K. Laakkonen

**Affiliations:** 1 Gerontology Research Center, Faculty of Sport and Health Sciences, University of Jyväskylä, Jyväskylä, Finland; 2 Department of Medical Biochemistry and Genetics, Institute of Biomedicine, University of Turku, Turku, Finland; 3 Turku Clinical Sequencing Laboratory, University of Turku, Turku, Finland; 4 Biocenter Oulu, University of Oulu, Oulu, Finland; 5 Department of Medical Rehabilitation, Oulu University Hospital, Oulu, Finland; 6 Center for Life Course Health Research, University of Oulu, Oulu, Finland; 7 National Institute for Health and Welfare, University of Helsinki, Helsinki, Finland; 8 Institute for Molecular Medicine FIMM, University of Helsinki, Helsinki, Finland

## Abstract

Exosomes participate in intercellular messaging by transporting bioactive lipid-, protein- and RNA-molecules and -complexes. The contents of the exosomes reflect the physiological status of an individual making exosomes promising targets for biomarker analyses. In the present study we extracted exosome microRNAs (exomiRs) from serum samples of premenopausal women (n = 8) and monozygotic postmenopausal twins (n = 10 female pairs), discordant for the use of estrogenic hormone replacement therapy (HRT), in order to see whether the age or/and the use of HRT associates with exomiR content. A total of 241 exomiRs were detected by next generation sequencing, 10 showing age, 14 HRT and 10 age +HRT -related differences. When comparing the groups, differentially expressed miRs were predicted to affect cell proliferation processes showing inactivation with younger age and HRT usage. MiR-106-5p, -148a-3p, -27-3p, -126-5p, -28-3p and -30a-5p were significantly associated with serum 17β-estradiol. MiRs formed two hierarchical clusters being indicative of positive or negative health outcomes involving associations with body composition, serum 17β-estradiol, fat-, glucose- and inflammatory markers. Circulating exomiR clusters, obtained by NGS, could be used as indicators of metabolic and inflammatory status affected by hormonal changes at menopause. Furthermore, the individual effects of HRT-usage could be evaluated based on the serum exomiR signature.

MicroRNAs (miRs) are a class of epigenetic regulators, small non-coding RNAs, which bind to their target mRNAs leading to RNA silencing and further to translational suppression. MiRs are localized in various cell types as well as in all body fluids. To date it has been shown that circulating miRs are either attached to vesicle free protein complexes, such as argonaute proteins (Ago 1–4)^1^,^2^ or high density lipoprotein (HDL) molecules^3^, localized inside the apoptotic bodies^4^ or exosomes^5^. Especially exosome miRs (exomiRs) are known to be relatively stable in the circulation.

Exosomes are small (<100 nm in diameter) spherical bilayer proteolipid vesicles produced by various cell types under both normal and pathological conditions. They bud from the late endosomes in the cytoplasm forming multivesicular bodies (MVB) and either remain in the cells they are formed or are fused with the plasma membrane and exported to the extracellular matrix or further to the circulation^6^. It has been shown that exosomes consist of lipids, proteins and different sized RNA molecules including miRs^5^,^7^. The content of the exosomes is affected by their source and also supposedly due to the physiological condition of the exosome secreting cell. Quite recent studies have demonstrated that the messages, delivered by the secreted exosomal RNAs, can generate changes in the gene expressions and protein concentrations of the recipient cells, suggesting the functionality of the exosome content in the target cells^5^. Especially the immuno-stimulating role of the exosomes has been emphasized e.g. by showing that dendritic cells communicate via so called exosome-miR shuttles in mice^8^.

Estrogens, female sex steroid hormones, have broad systemic effects on different cell types through their receptors. As known, the levels of systemic estrogens, especially 17β-estradiol (E_2_), change dramatically at the time of menopause in women. Estrogen sensitivity of miR regulation and their expression in different cell types has been recently demonstrated in several studies^9^,^10^,^11^,^12^,^13^. Our previous studies have shown that circulating cell free miRs, including miR-21 and miR-146a differ between postmenopausal genetically identical twin sisters who are discordant for the estrogen based hormone replacement therapy (HRT) suggesting that systemic estrogen levels affect miR profile^14^.

A very recently published study demonstrated an association between accelerated aging, measured by the epigenetic age i.e. DNA methylation status of blood, and early onset of menopause^15^. In the present study, we investigated whether the age or/and the use of postmenopausal HRT associates with the miR content of the circulating exosomes. We extracted exosomes from the serum of healthy premenopausal women (n = 8) not using external estrogen products and from healthy postmenopausal monozygotic (MZ) twin sisters discordant for the use of HRT (n = 10 pairs). Next generation sequencing (NGS) was used to analyze the exomiR content and CAP-miRSEQ pipeline together with Poisson-normal regression model to identify specific miRs with differential expression pattern. Our results indicated that specific miRs differ in terms of the age of the sample donator and HRT treatment. These miRs have been shown to function as regulators of cellular homeostasis. Hierarchical clustering analysis revealed two miR-clusters, the other indicating negative association with E_2_ parallel to positive association with markers of adiposity and inflammation and the other being opposite in the associations with E_2_ and metabolic health markers. These results suggest that female E_2_ status may be a mediator of the change in exomiR content that in turn is associated with change in health metabolic status known to occur within menopause.

## Results

### Participant characteristics

Participant anthropometrics and measured blood characteristics as well as group differences are presented in the Table 1. All groups differed significantly from each other in serum E_2_ and follicle stimulating hormone (FSH) levels those being highest (E_2_) and lowest (FSH) among premenopausal women as expected. Waist circumference was significantly lower among premenopausal women compared to both postmenopausal groups. Fat percentage was significantly lower in postmenopausal HRT users compared to their co-twins. Serum TNF-α and plasma glucose concentrations were also significantly lower among premenopausal women in comparison to postmenopausal women.

### ExomiR profile and differentially expressed miRs in NGS

**Figure 1:**
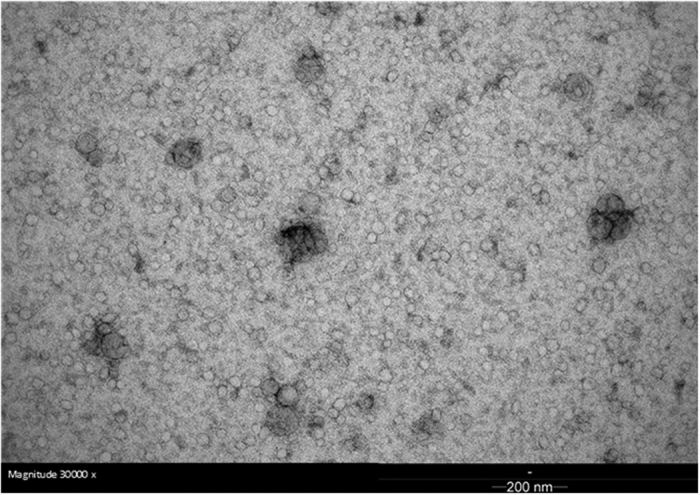
Electron microscope image of the extracted serum vesicles. Typical size of an exosome is less than 100 nm in diameter. A Tecnai G2 Spirit transmission electron microscope (FEI, Eindhoven, The Netherlands).

**Figure 2:**
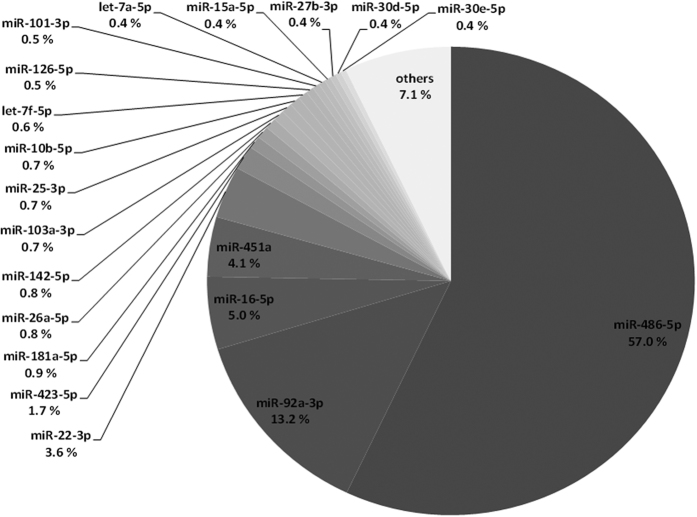
Relative serum exomiR content of all the samples.

The functionality of the used exosome extraction method was demonstrated by electron microscope imaging (Fig. 1, a representative image obtained from the serum exosomes of a postmenopausal HRT user). Most of the detected lipid vesicles were in normal range for exosomes being under 100 nm in diameter. The exosome quantity, measured by esterase activity, showed no differences between the studied groups (Pre = 17566 ± 1196 RFU/μg, HRT = 17876 ± 5801 RFU/μg, No HRT = 16303 ± 4370 RFU/μg, P = 0.610–0.925). Altogether, 241 different known miRs were detected in the serum exosomes by NGS. The processed sequencing reads are presented in Supplementary data (S1). The most abundant miRs of the exosome cargo include miR-486-5p, -92a-3p, -16-5p, -451a, -22-3p and -423-5p together covering up to 84.6% of the exomiR content (Fig. 2).

The miR read counts from the differentially expressed miRs are presented in Table 2. Altogether, 21 miRs had significant differential expression pattern (p-value with false discovery rate (FDR) correction, P < 0.05) in NGS either in one, two or three group comparisons (postmenopausal No HRT group vs. premenopausal group, postmenopausal HRT-user group vs. premenopausal group or postmenopausal HRT-users vs. postmenopausal non-using co-twins). MiR-126-5p, -142-5p, -484 and -10b-5p were differentially expressed between all the studied groups *(P* < *0.001)* showing age- and HRT-use associated differences. MiR-27b-3p, -10a-5p, -215-5p and -144-5p were differentially expressed between both postmenopausal groups vs premenopausal women showing age-associated differences with or without HRT (P < 0.001). MiR-148-3p and -28-3p were differentially expressed between postmenopausal No HRT group vs premenopausal women (*P *=* 0.008, P *=* 0.009, respectively*) and miR-375 and -186-5p between postmenopausal HRT group vs premenopausal women (*P *=* 0.045, P* < *0.001, respectively*). MiR-532-5p, -1285-3p, -30a-5p, -3688-3p, -29b-3p, -106b-5p, -29c-3p, -1306-5p, -148a-3p and -301a were differentially expressed between HRT users and their non-using co-twins suggesting association between HRT- use and miR expression (*P* < *0.001, P* < *0.001, P *=* 0.001, P *=* 0.016, P *=* 0.029, P *=* 0.029, P *=* 0.033, P *=* 0. 036, P *=* 0.049, P *=* 0.049, respectively)*.

### Functional network analysis

**Figure 3:**
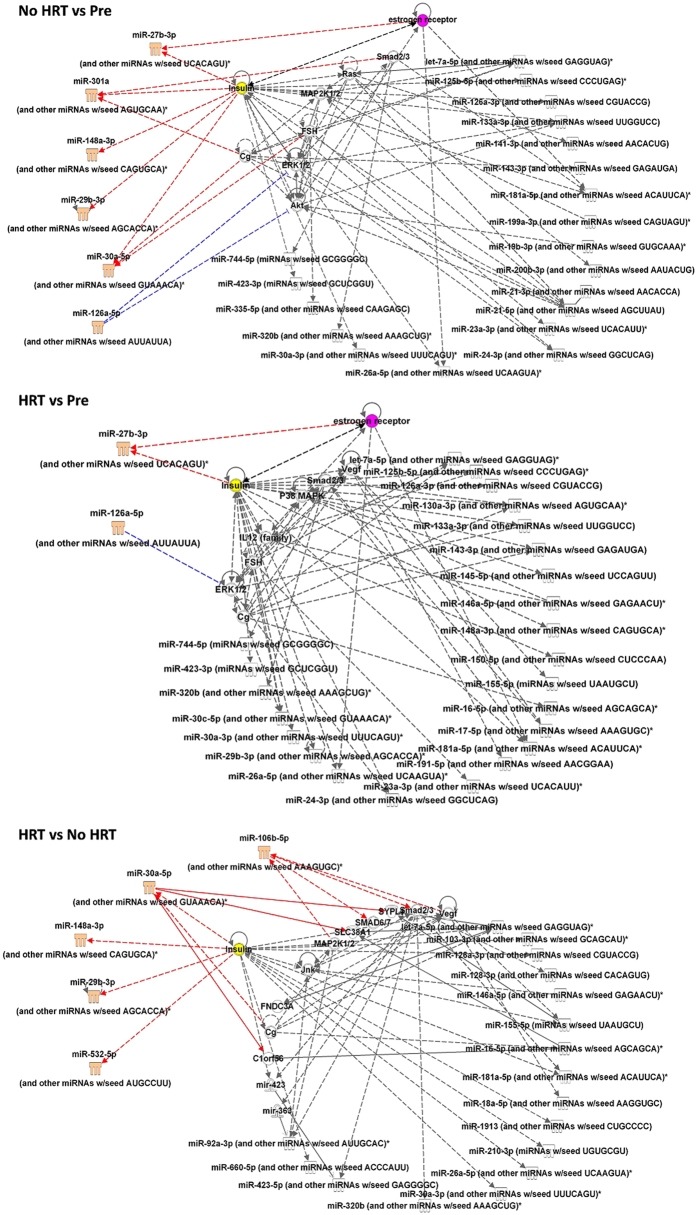
The most prominent network from IPA comparison analyses performed to all sequenced miRs. Differentially expressed miRs (FDR) are highlighted in the figure with coral colour. The arrow head of the red line tells the direction of the predicted affect. Blue line represents the predicted inhibition. Insulin is highlighted in yellow and ESR in pink. Smad2/3: Smad family member 2 or 3, Ras: protein superfamily of small GTPases, MAPK2K1/2: Mitogen activated protein kinase 2K1 or 2, FSH: Follicle stimulating hormone, Cg: Chorionic gonadotrophin, VEGF: Vascular endothelial growth factor, IL12: Interleukin 12, ERK1/2: Extracellular signal regulated kinase 1 or 2, SYPL1: Synaptophysin like 1. SLC38A1: Solute carrier family 38 member 1, Clorf56: Chromosome 1 open reading frame 56 solute, Jnk: Jun kinase, FNDC3A: fibronectin type III domain containing 3A.

All sequenced miRs were included in the analyses to identify the most prominent networks by explorative comparison analyses by Ingenuity pathway tools (IPA) (Fig. 3). No cut-offs for p-values were used. The age comparison between postmenopausal women without HRT and premenopausal women suggested that estrogen receptors (ESRs) and insulin are predicted to be the biggest upstream regulators of the miRs in the network. Six of the differentially expressed miRs (FDR <0.05) listed in Table 2 were involved in the network and highlighted in the figure. The comparison between postmenopausal HRT users and premenopausal women involved also ESRs and insulin as upstream regulators but only two of the differentially expressed miRs, miR-27b-3p and miR-126-5p. The comparison between the postmenopausal twin sisters involved five of the differentially expressed miRs out of which four are predicted to be regulated by insulin. In addition, miR-106b-5p was predicted to have VEGF and SMAD6/7 as upstream regulators and miR-30a-5p chorionic gonadotropin (Cg).

**Figure 4:**
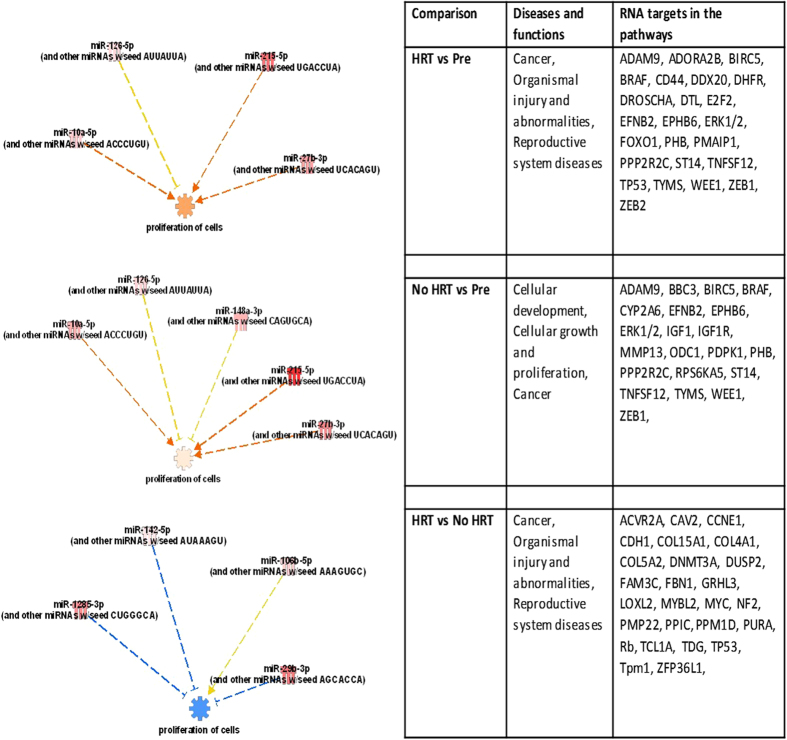
IPA comparison analysis of the 3 different comparisons for differentially expressed miRs. The figure shows miRs in each comparison predicted to regulate cell proliferation. Red color indicates activation, blue inhibition and yellow controversial findings of the specific miR. Younger age and the use of HRT were predicted to inhibit the cell proliferation. The table shows the main networks for differentially ex-pressed miRs identified by IPA with its annotation as the top diseases and functions and the interacting molecules i.e., potential miR targets as well as miRs included in the network. The underlined miR targets are also involved in cell proliferation (figure).

To focus on the most significant miRs, comparison analysis was performed to differentially expressed miRs (cut-off P < 0.05) by IPA, including all the three group comparisons (Fig. 4). Cell proliferation was the most significant downstream target process predicted to be affected in all the comparisons. Comparisons between premenopausal and both postmenopausal groups included the same miRs (miR-27b-3p, -215-5p, -126-5p and -10a-5p), except for miR-148a-3p which was included only in the premenopausal vs postmenopausal No HRT group network. Higher age (or postmenopausal status) was predicted to up-regulate the cell proliferation pathway whether using HRT or not. Other set of miRs (miR-29b-3p, -106b-5p, -142-5p, and -1285-3p) were shown to have an effect when comparing the postmenopausal co-twins with each other. In this comparison, the use of HRT was predicted to down-regulate the cell proliferation pathway. Figure 4 shows the main networks for differentially expressed miRs identified by IPA with its annotation as the top diseases and functions and the interacting molecules i.e., potential miR targets as well as miRs included in the network.

### Associations of NGS reads with serum E_2_ and other variables

**Figure 5:**
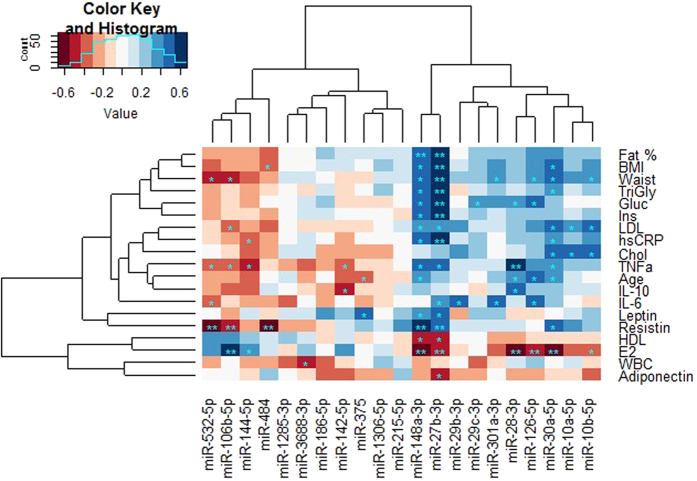
Clustered heatmap of the selected miRs and other measured variables. Blue indicates positive and red negative associations. Spearman correlation coefficient was used for the analyses. *P < 0.05 (nominal), **P < 0.05 (FDR corrected). Positive health outcome cluster is on the left side with positive associations with E2 and negative health outcome cluster on the right side with negative E2 associations. BMI: body mass index, TriGly: triglycerides Gluc: plasma glucose, Ins: serum insulin, hsCRP: high sensitive c-reactive protein, IL-6: interleukin 6, LDL: low densi-ty lipoprotein, TNFa: tumour necrosis factor alpha, IL-10: interleukin 10, Chol: cholesterol, WBC: white blood cell count, HDL: high density lipoprotein, E2: 17β-estradiol. RStudio Team (2015). RStudio: Integrated Development for R. RStudio, Inc., Boston, MA URL http://www.rstudio.com/.

The associations of the differentially expressed miRs with serum E_2_ concentration, body composition and fat-, glucose- and inflammation related markers are visualized as a correlation heatmap (Fig. 5). The p-values, FDR-values and correlation coefficients are presented in Supplementary data (S2). Heatmap reveals hierarchically clustered information about the associations of the selected miRs with other measured variables. First of all, six miRs from the differentially expressed NGS results were significantly (FDR < 0.05) associated with serum E_2_. MiR-106b-5p was positively associated with serum E_2_ concentration, while other miRs, miR-148a-3p, -27-3p, -126-5p, -28-3p and -30a-5p were negatively associated with serum E_2_ concentration. In addition, miR-144-5p had indicatively significant positive association and miR-10b-5p indicatively significant negative association with E_2_ concentration (P = 0.025, FDR = 0.066 for both associations). Although only the six mentioned miRs possessed significant association with FDR < 0.05, it is clear from the heatmap that the miRs under investigation form two separate clusters the other cluster being indicative of positive and the other of negative health outcomes.

The positive health outcome cluster miRs had positive associations with E_2_ and the negative health outcome cluster miRs had negative associations with E_2_ and concomitant negative and positive associations, respectively, with most of the adiposity and inflammation related variables except that with HDL and adiponectin which were clustered together with E_2_ concentration. However, the associations with miRs and HDL count were only indicatively significant for miR-148a-3p and -27b-3p with HDL (P = 0.009, FDR = 0.126 and P = 0.012, FDR = 0.126, respectively) and for miR-27b-3p with adiponectin (P = 0.013, FDR = 0.273) while for the other miRs the associations were non-significant. The leading miRs for the negative health outcome cluster were miR-27b-3p, which had the highest number of significant associations and miR-148a-3p, which had very similar profile although some of the associations were only nominally significant. The higher NGS read count value of miR-27b-3p was significantly associated (FDR < 0.05) with greater adiposity (fat percentage, BMI, waist circumference, triglyceride concentration), with higher fasting glucose and insulin and with higher hsCRP, resistin and TNF-α (P = 0.014, FDR = 0.095) together with lower E_2_ concentration. The positive health outcome cluster did not have as prominent leading miR, but the miRs having positive association with E_2_, miR-106b-5p and miR-144-5p, had also negative association with TNF-α (P = 0.027, FDR = 0.099 and P = 0.008, FDR = 0.084, respectively). In addition, miR-106b-5p, -532-5p and -484 had statistically significant (FDR < 0.05) negative associations with resistin.

### Validation of differentially expressed sequenced miRs

**Figure 6:**
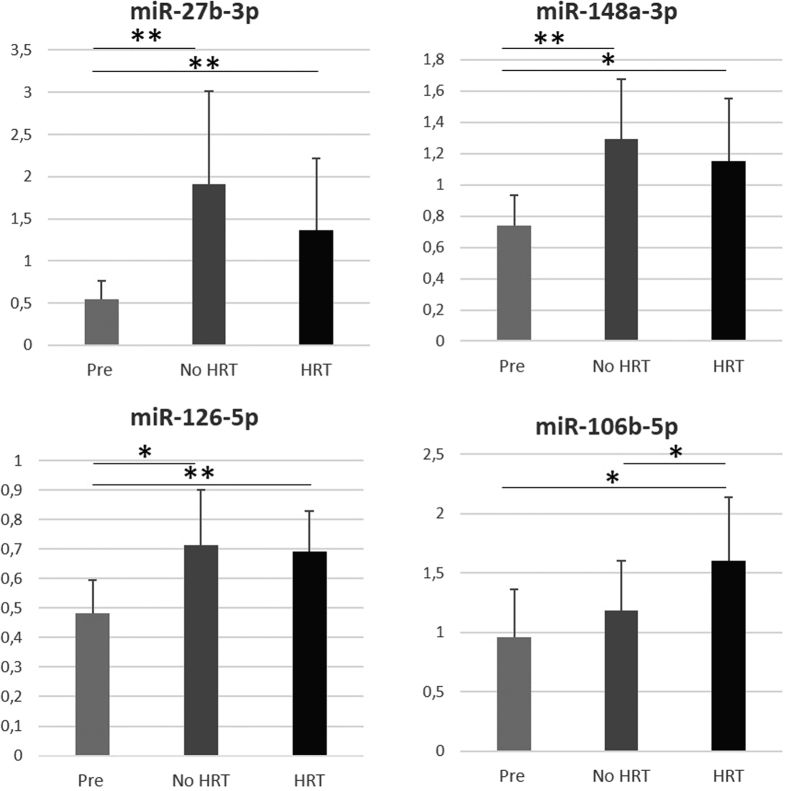
qPCR validation of the miRs associated with systemic E2. Pre: Premenopausal women, No HRT: Postmenopausal women without hormone replacement therapy, HRT: Postmenopausal women using HRT. *P < 0.05, **P < 0.01. Results are presented as mean relative ex-pressions +SD.

qPCR validations of the E_2_-associated miRs are presented in Fig. 6. For miR-27b-3p, -148a-3p and -126-5p the levels were significantly lower in premenopausal women compared to postmenopausal No HRT group (P = 0.001, P = 0.001, P = 0.040, respectively) and HRT group (P = 0.005, P = 0.040, P = 0.005, respectively). MiR-106b-5p was significantly lower in premenopausal women compared to postmenopausal HRT group (P = 0.040) and lower in No HRT women compared to their HRT co-twins (P = 0.043). No differences between any groups were detected for miR-28-3p and -30a-5p (data not shown).

## Discussion

This is the first study showing that age and the use of HRT are associated with the miR contents of the circulating exosomes in women. Altogether, 241 distinct known serum exomiRs were detected by NGS among the studied groups from which 21 were differentially expressed. The NGS results showed that exomiR -levels differ between all of the three studied groups significantly in cases of four miRs. In addition, age-associated difference without HRT was detected in six miRs (postmenopausal No HRT vs premenopausal), and with HRT in ten miRs (postmenopausal HRT vs premenopausal) as well as treatment difference in ten miRs (postmenopausal HRT vs No HRT).

Menopause is recognized as a risk factor for the development of metabolic dysfunctions^16^,^17^. The loss of circulating E_2_ is followed by androgen dominance and further by body fat accumulation around the abdominal area^18^. The excess amount of adipose tissue leads to unbalanced cytokine profile which is seen as increased levels of IL-6, TNF-α, IL-1β, resistin and leptin among other inflammatory molecules^19^. These circulating pro-inflammatory cytokines form a base for systemic low-grade inflammation also known to be associated with aging^20^. In addition to adipose tissue initiated release of pro-inflammatory cytokines, also issues in glucose metabolism, such as insulin resistance play a role in the systemic inflammation^21^. Furthermore, endothelic dysfunction and atherosclerosis are adding to the chronic inflammatory state^22^. In our study the premenopausal women had healthier profile in terms of metabolic measures such as waist circumference and plasma glucose levels compared to postmenopausal women. In addition, fat percentage was significantly higher among the postmenopausal women who were not under HRT compared to their co-twins under HRT. We were able to see a slight increase in TNF-α levels due to age, whether under HRT or not, but no significant differences in other classical pro-inflammatory markers between any of the studied groups. However, across the measured inflammatory markers a trend towards the most unbeneficial state was detected among postmenopausal women, who were not under HRT. Clear expected differences were detected in systemic steroid hormone levels as well as gonadotropins, from which high FSH levels are indicators of postmenopausal state. In addition, interesting associations with serum E_2_ concentrations and differentially expressed miRs, inflammation, glucose metabolism and adiposity -related markers were found.

The present study revealed six different miRs which were associated (FDR < 0.05) with serum E_2_ levels either positively (miR-106b-5p) or negatively (miR-148a-3p, -27-3p, -126-5p, -28-3p and -30a-5p). In addition, two miRs had indicatively positive (miR-144-5p, P < 0.05) and negative (miR-10b-6p, P < 0.05) association with serum E_2_. To our knowledge two of these miRs have been previously shown to have an association with E_2_ in breast cancer cells. Nassa *et al*.^23^ have demonstrated that miR-30a-5p is regulated by estrogen receptor β (ESRβ) whereas Tao *et al*.^24^ demonstrated that miR-148a is downregulated by E_2_ through GPER in breast cancer cells. In addition, 17-β-estradiol–ESRα–miR-27b -connection has been demonstrated on human leukemia cell line^25^. Our NGS data indicated significant differences between postmenopausal twins for miR-30a-5p and between all the studied groups for miR-148a-3p and miR-27b-3p. All miRs had a negative association with serum E_2_ which is in line with the mentioned studies.

Based on recent publications, some of the E_2_ associated miRs identified in the present study have connections to aging. MiR-10b-5p has been shown to be associated with the motor onset in both Parkinson’s and Huntington’s diseases^26^ indicating its function in the development of age-associated neurodegenerative disorders. Also, the role of circulating miR-10b-5p in osteogenic differentiation after fracture at postmenopausal age has been recognized^27^. In addition, changes in miR-28-3p levels during early senescence were observed in endothelial cells, thus, indicating miR’s possible regulatory role in the aging of vascular endothelium. Even though no direct effect of E_2_ has been stated in the mentioned studies, it has been well demonstrated that estrogen has a role as a neuro-, osteo- and vascular protector^28^,^29^,^30^,^31^. Our earlier studies have also shown beneficial effects of postmenopausal HRT on body composition measures, including fat, muscle and bone properties^32^,^33^,^34^,^35^,^36^. The connection between lipoproteins, vascular inflammation and exosomes has also been recognized by others^37^. MiR-27b-3p has been shown to regulate fat metabolism and inflammation by targeting RXRα and PPARγ^38^,^39^. In the present study, mir-27b-3p was one of the most interesting single miRs belonging to the negative outcome cluster. The NGS data indicated higher levels of mir-27b-3p in the circulating exosomes of the postmenopausal twins compared to premenopausal women indicating an age association. This miR was negatively associated with serum E_2_, HDL and adiponectin (last 2 nominal) whereas positive associations were detected with other adiposity markers such as fat percentage and triglycerides and nominally with LDL. In addition, the levels of miR-27b-3p were positively associated with serum hsCRP and resistin as well as insulin and plasma glucose concentrations, all of these markers indicative of unbeneficial metabolic and inflammatory status. The associations for miR-148a-3p were similar to miR-27b-3p but only nominally significant in some cases. Altogether, these results support the function of miR-27b-3p (and miR-148a-3p) in the regulation of lipid and glucose metabolism and further reveal its possible negative role in women’s aging with hormonal changes.

Different miR carrier systems share a bunch of common miRs but they also seem to have a unique content which differs from the other miR vehicles and, also, from the cell of origin^40^. Exosomes have been shown to deliver gene-based intercellular messages adding to the complexity of cell-cell communication. According to Cheng *et al*.^41^, exosomes provide a protected and enriched environment for miRs compared to the intracellular and other cell free miRs. In their study, exosome derived miRs were associated with neuronal signaling whereas the main responsibility of the cell free miRs was the signaling related to the cellular homeostasis, emphasizing the functional differences between cell free miRs and exomiRs. In our previous studies, we have shown that the circulating cell free levels of miR-21 and miR-146a differ between the HRT users and their non-using co-twins^14^. In the present study, we were able to detect the same miRs in the exosomes by NGS, however, significant differences were not obtained. That may be considered as a supporting indicator of the differential roles of the two types of miR transfer. The mechanism how the exomiRs are sorted into the exported exosomes is an unanswered question. It is under active speculation whether the sorting takes place by the miR sequence or whether the exomiR content is a description of the whole miR pool of the exosome forming cell. It is important to recognize that the sequencing data can be interpreted by several ways: one could either focus on the profile as a whole and compare the relative amounts and changes in the relative patterns, or the other option is to focus on specific miRs. Validation of specific miRs is not inevitably the whole truth as very often miRs work as a group fine-tuning each other’s function. In the present study, qPCR validation of the six E_2_-associated miRs confirmed the NGS results fully for miR-27b-3p and miR-126-5p and partly for miR-148a-3p and miR-106b-3p, however, no differences for miR-28-3p and -30a-5p were detected.

According to the functional network analyses obtained by IPA, the most prominent predicted upstream regulators of the studied miRs include insulin as well as estrogen receptors. These results support the link between the systemic E_2_ levels, insulin signaling and the studied miRs. In the comparison analyses performed using only the differentially expressed miRs, the cell proliferation processes were predicted to be inactivated at younger age as well as due to the use of HRT. Interestingly, different set of miRs seem to regulate cell proliferation processes depending on whether comparing only postmenopausal twins or premenopausal and postmenopausal women. Cell proliferation is usually considered to be more active at younger age. However, as we do not know the cell origin or destination of the exomiRs, we cannot say much about the specific processes and cells under the influence. Based on the present findings, the predicted activation of cell proliferation especially among the postmenopausal women without HRT, could be related to increasing amounts of adiposity, inflammatory cells or possibly tumor cells, all related to natural aging. These results suggest that the postmenopausal HRT users are more similar to premenopausal women in terms of miR regulated cell proliferation than postmenopausal non-users. Based on this genetically controlled study, these effects are achieved to most part by the HRT usage. The study has been performed with a small unique population and thus cannot be generalized directly to female population as a whole. However, as a MZ twin study, it provides a valuable genetically controlled design. Further cell culture experiments are needed in order to say more about the specific functions of HRT sensitive exomiRs on specific cell types.

The recent achieved technologies in molecular biology, including NGS, provide promising tools to discover the broad functions of miRs in both physiological and pathological conditions. To our knowledge, the present study was the first to show a detailed serum exomiR profile of women with different age and hormonal status. We demonstrated that the differential expression patterns were emphasized among exomiRs interplaying with cellular homeostasis, glucose- and lipid metabolism as well as inflammation. Furthermore, we were able to identify miR groups related to more positive or negative health outcomes in which the systemic E_2_ concentration played a significant role as a divider. Our findings suggest that the serum exomiRs are sensitive to hormonal changes among women and carry important regulatory messages between cells. The results can be used as a directional starting point for the usage of miR signature, obtained by NGS, as a medical tool for prognostics and diagnostics in aging women. In addition, the miR signature can potentially be applied when evaluating the benefits of HRT usage and its individual suitability in personalized medicine.

## Materials and Methods

### Study design

The present study is part of the research project “Sarcopenia and Skeletal Muscle Adaptation to Postmenopausal Hypogonadism: Effects of Physical Activity and Hormone Replacement Therapy in Older Women–a Genetic and Molecular Biology Study on Physical Activity and Estrogen-related Pathways (SAWEs)”. A more detailed design and the recruitment process of the SAWEs- study has been described previously^42^,^43^. Briefly, the study participants were recruited from the Finnish Twin Cohort (n = 13888 pairs)^44^. The invitation was sent only to women born in 1943-1952 (n = 537 pairs). To be able to take part in the study, both co-sisters needed to participate. This study included 15 MZ female twin pairs who had no contraindications, were discordant for the use of HRT (mean duration of HRT use 6.9 ± 4.1) and were willing to participate to the study. Five of the HRT users were using preparations containing only E_2_ (1-2 mg), six used estrogenic (1–2 mg)+ progestogenic compounds and four tibolone (2.5 mg) based treatment. Since the aim of the present study was to investigate the effects of E_2_ based HRT, tibolone based HRT users and their co-twins were excluded. Finally, 11 MZ twin pairs were included in the present study, however, the NGS read quality of one sample was not sufficient enough thus, this twin pair was excluded from the analysis. The number of participants is relatively small but comparable with other MZ co-twin studies^45^,^46^,^47^ and has enough statistical power to obtain clinically relevant results. No significant differences in physical activity levels (slightly modified Grimby scale^48^), daily energy intake (5-day diary) or smoking habits between the twins were identified. In addition, eight women aged 30 to 40 years with no use of hormonal contraceptives during the last 5 years, were included for the present study representing a premenopausal group. Contraindications for participation were chronic musculoskeletal diseases, type 1 or 2 diabetes, mental disorders, asthma with oral glucocorticosteroid treatment, cancer, drug or alcohol abuse, and Crohn’s disease. High blood pressure was the only condition to which daily medication was used (HRT: n = 3, No HRT: n = 4). Other occasional medication included antihistamines, paracetamol and ibuprofein. Overall, the participants were considered as healthy women.

The study was conducted according to the guidelines of the Declaration of Helsinki and the protocol was approved by the Ethics Committee of the Central Finland Hospital District (E0606/06). Written informed consent was provided by the study participants prior the laboratory measurements.

### Serum analyses

Whole blood samples were collected under standard fasting conditions from antecubital vein in a supine position. The samples of the premenopausal women were collected during the first five days of the menstrual cycle representing the lowest E_2_ concentrations. The blood was allowed to clot for 30 mins in room temperature followed by serum separation by centrifugation at 4000 rpm. All the samples were snap frozen and stored in −70 °C in 0.5 ml aliquots.

### Exosome isolation, imaging and RNA extraction

Serum exosomes were isolated from 450 μl of sample by using Exoquick Exosome Precipitation Solution according to manufacturer’s protocol (#EXOQ5A-1, System Biosciences).

Electron microscopy was used for checking the size of the extracted vesicles. Briefly, the isolated exosomes were deposited on Formvar carbon-coated, glow-discharged grids. After 20 minutes, the grids were washed with PBS and exosomes were fixed in 1% glutaraldehyde for 5 minutes. After washing with distilled water the grids were stained with neutral uranylacetate and embedded in methylcellulose/uranyl acetate and examined in a Tecnai G2 Spirit transmission electron microscope (FEI, Eindhoven, The Netherlands). Images were captured by Quemesa CCD camera using iTEM software (Olympus Soft Imaging Solutions GMBH, Munster, Germany). FLUOROCET Ultrasensitive Exosome Quantitation Assay Kit (System BioSciences), measuring exosome esterase activity, was used to assess the exosome quantity in samples (4 per each studied group) according to manufacturer’s protocol. Briefly, the protein concentration of exosome-PBS -solution was measured using BCA protein method by Nanodrop (1000) using wavelength 562 nm. Equal volume of exosome preparation was loaded into each 96-well plate. Promega Glomax Multi + Detection system with fluorescence module was used for the measurement (excitation = 525 nm, emission = 580 to 640 nm). Results are presented as relative fluorescence unit (RFU) per total protein content of the sample (RFU/μg).

Total RNA extraction was performed by using Trisure reagent (Bioline) according to manufacturers’ instructions with slight modifications. Additional step was added to the homogenization step where 7 μl of synthetic cel-miR-39 miR mimic (1,6 × 10^8^ copies/μl, Qiagen cat. no 219610) was added to each sample to serve as a spike-in control for monitoring the miR purification and amplification. Chloroform was used for the phase separation and 1 μl of nuclease free glycogen (Glycogen RNA Grade, 20 mg/ml, Fermentas) for enhancing the RNA precipitation. RNA concentration was measured by Nanodrop 1000 (Thermo Scientific). Prior the library preparation RNA quality and recovery was checked by qPCR according to manufacturer’s protocol (Qiagen miScript Primer assays and II RT kit for cDNA synthesis and MiScript SYBR Green PCR Kit for RT-qPCR) from which the recovery of cel-miR-39 spike-in control (Ct_mean_ = 24.9 ± 1.0) and miR-21-5p (Ct_mean_ = 27.3 ± 1.3) was verified.

### cDNA library preparation and small RNA sequencing

The small RNA libraries were prepared using TruSeq Small RNA Sample Preparation Kit (Illumina, USA) with multiplexing adapters. Following the TruSeq Small RNA Sample Preparation Kit user guide (Rev. E), the total RNA, including the small RNA fractions, were ligated to 5′ and 3′ adaptors sequentially before converted to cDNA by reverse transcription. cDNAs were amplified with PCR by using primers containing unique six base index sequences distinguishing different samples from one another. Finally, the samples were subjected to 6% (w/v) non-denaturing polyacrylamide gel electrophoresis (PAGE). cDNA library fragments between 145 and 160 bp corresponding the miR libraries were excised from the gel, purified and eluted. The final miR library pellet was air dried and resuspended in 10 μL nuclease-free water and quantity of the libraries was measured with Qubit fluorometer. Ready miR library pools (8–16 samples on single pool) were loaded to MiSeq V3 flow cell in 12 pM concentrations. To increase signal integrity, 10% of PhiX was spiked in the library pool. MiR libraries were sequenced with MiSeq reagent kit V3 150 cycles using 36 bp reads with single-end chemistry. Three replicates were used in each NGS run.

### miR validation

The miRs having differential expression in NGS results and significant association with systemic E_2_ (FDR < 0.05, Fig. 5) were validated with RT-qPCR. These miRs included miR-27b-3p, -148a-3p, -126-5p, -28-3p, -30a-5p and -106b-5p. Validation was performed using samples from five postmenopausal twin pairs and ten premenopausal women. MiScript II RT Kit was used for cDNA synthesis (Qiagen) according to manufacturer’s protocol. cDNA was diluted 1:3 for the RT-qPCR which was performed using miScript SYBR Green PCR Kit and miScript Primer assays (Qiagen: Cat. No. MS00031668, MS00003556, MS00006636, MS00009254, MS00007350, MS00003402, MS00019789) according to manufacturer’s protocol. Ct-values less than 36 were included in the analyses. Results were normalized to spike-in cel-miR-39 values of each sample (Ct(av) = 25.1 ± 1.0). A pooled calibrator sample was used across the different plates to obtain 2^−dCt^ (=RQ) results. Subsequently, normalized relative quantities (NRQ) were calculated for each miR of each sample (NRQ = RQ/NF), where normalization factor (NF) presented the geometric mean of RQs of all expressed miRNAs per sample^49^.

### Bioinformatics and statistical analyses

**Figure 7:**
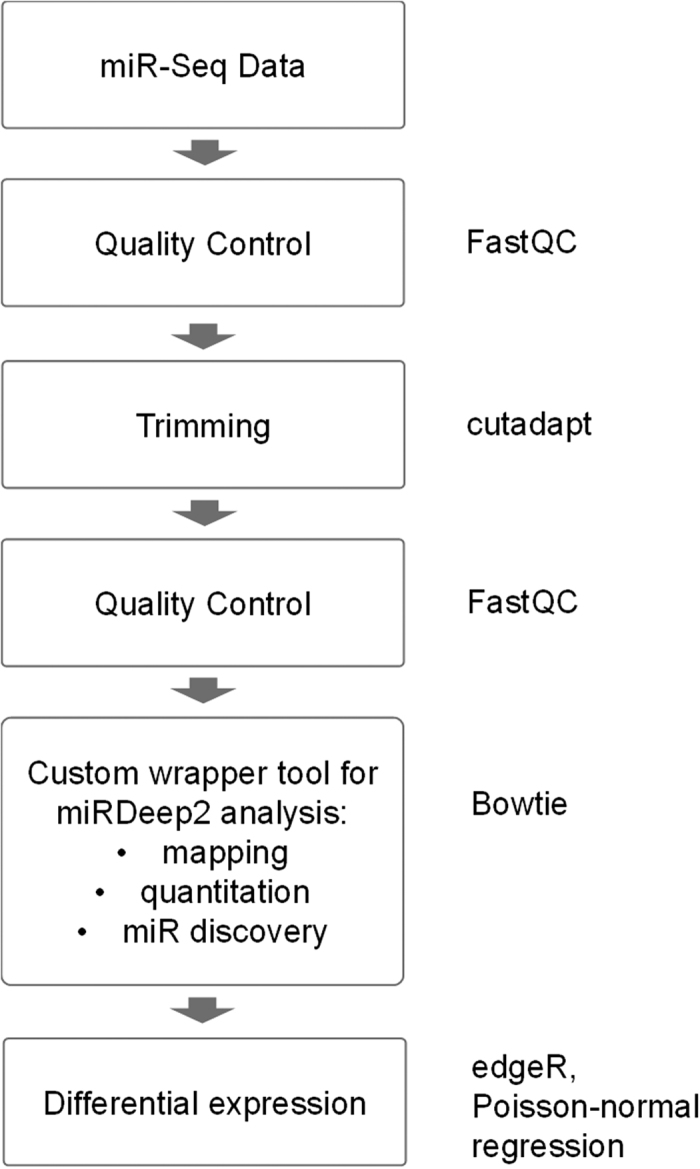
Workflow of the handling of the sequencing data. All the methods and R packages used in the analyses are presented in the figure.

The workflow for cleaning and analyzing the sequencing results is presented in the Fig. 7. Briefly, quality of the raw reads was assessed with FastQC and reads were trimmed using cutadapt based on the FastQC ‘Overrepresented sequences’ module output and using a minimum read length filter of nine bases. Trimmed read files were analyzed with miRDeep2, a comprehensive computational tool for miR analysis and discovery which uses a probabilistic model of miR biogenesis to score compatibility of the position and frequency of sequenced RNA with the secondary structure of the miR precursor^50^. Mapping to the reference genome (hg38) was performed using bowtie (version 1.0.1) and miRbase version 18 was used for retrieving miR information. Differential expression analysis was performed on miRDeep2 output data utilizing a custom R script adapted from the differential expression module in the CAP-miRSEQ tool^50^,^51^. The tools employed in different steps rely on various programming languages. To simplify the workflow a set of R functions was created to carry out QC, trimming, miRDeep analysis and assessment of differential expression in a pipeline-like fashion run from one wrapper script. In addition, Poisson-normal regression model was created to analyze the related participants (unpublished manuscript). Briefly, read counts were modeled as Poisson distributed variables with over distribution modeled through a normally distributed random variable. The advantage of this model over e.g. the negative-binomial model is that it allows flexible modeling of dependency among related subjects through the random effect correlation matrix. The model was applied on each miR on MPlus version 7 and FDR approach was used to adjust for multiple testing. All other statistical computations were done in R software (versions 3.1.3–3.3.1). R packages used in the bioinformatics workflow are mentioned in Fig. 7.

The group analyses are based on three groups: (1) premenopausal women (n = 8), (2) postmenopausal No HRT (n = 10) and (3) postmenopausal HRT users (n = 10), the latter two forming MZ twin pairs. Based on the normal distribution of the studied variables, tested by Shapiro-Wilk test, the group comparisons were performed either with Independent samples T test and paired samples T test (twins), in case of parametric variables, or with Mann Whitney U test and Wilcoxon signed-rank test (twins) in case of non-parametric variables. Two-sided tests were used. Ingenuity pathway analyses (IPA) tool was used for pathway and comparison analyses of all sequenced miRs and differentially expressed miRs. P < 0.05 was considered significant. Spearman’s rank correlation coefficient was used for correlation analyses and R packages “gplots” and “RColorBrewer” were used for creating the clustered heatmap (Fig. 5). Data analyses and visualizations were carried out using Eclipse IDE Luna (4.4.2) and the StatET plugin for R (3.4.2) with RJ 2.0, IBM SPSS Statistics (version 23.0, Chicago, IL) and R Studio (R Studio Team 2015, Boston, MA).

### Data availability

The processed read counts are provided in the Supplemetary data (S1). The raw sequence read files are open at ArrayExpress database (www.ebi.ac.uk/arrayexpress) upon publication under access number E-MTAB-5245. Computer code availability is provided on request.

### Limitations

The methods of sample collection, RNA extraction and further analyses are not yet standardized in NGS. The enzymatic steps in small RNA cDNA library preparation might favor certain miRs over others due to sequence-specific biases. However, same protocol was used across the study, therefore such bias would occur similarly in each sample and would not affect the results of the group comparisons. Also exosome extraction methods lack standardization.

## Additional Information

**How to cite this article**: Kangas, R. *et al*. Aging and serum exomiR content in women-effects of estrogenic hormone replacement therapy. *Sci. Rep.* **7**, 42702; doi: 10.1038/srep42702 (2017).

**Publisher's note:** Springer Nature remains neutral with regard to jurisdictional claims in published maps and institutional affiliations.

## Supplementary Material

Supplementary Datasets

## Figures and Tables

**Table 1:** Participant body anthropometrics and blood characteristics and differences between the studied groups.

	PREMENOPAUSAL (N = 8)	POSTMENOPAUSAL NO HRT (N = 10)	POSTMENOPAUSAL HRT (N = 10)	SIGNIFICANT COMPARISON (P < 0.05)
Age (yrs)	32.0 ± 1.6	57.5 ± 2.0	57.5 ± 2.0	a, b
Height (cm)	164.5 ± 4.0	162.2 ± 4.5	162.8 ± 4.7	
Weight (kg)	70.0 ± 11.3	74.7 ± 15.6	67.8 ± 9.1	
BMI	25.9 ± 4.5	28.6 ± 6.8	25.7 ± 4.0	
Waist (cm)	86.4 ± 10.4	104.7 ± 9.6	100.9 ± 6.4	a, b
Fat %	29.7 ± 7.0	35.3 ± 9.3	30.5 ± 7.5	c
hsCRP (mg/l)	0.88 ± 1.1	1.53 ± 0.94	1.09 ± 0.95	
IL-10 (pg/ml)	0.89 ± 1.7	2.50 ± 3.70	2.43 ± 3.17	
IL-6 (pg/ml)	1.05 ± 0.70	1.77 ± 1.29	1.39 ± 0.71	
TNFa (pg/ml)	6.1 ± 1.6	10.5 ± 2.3	10.7 ± 4.8	a, b
WBC (e9/l)	6.1 ± 1.2	5.2 ± 1.4	5.3 ± 1.3	
RBC (e12/l)	4.5 ± 0.2	4.3 ± 0.3	4.4 ± 0.3	
HGB (g/l)	139 ± 5.0	137 ± 9.4	140 ± 9.1	
Plt (e9/l)	242.8 ± 54.2	233.9 ± 39.1	236.8 ± 83.4	
Chol (mmol/l)	4.7 ± 0.8	5.3 ± 0.4	5.3 ± 0.7	
LDL (mmol/l)	2.7 ± 0.8	3.1 ± 0.4	3.1 ± 0.8	
HDL (mmol/l)	1.6 ± 0.4	1.6 ± 0.4	1.6 ± 0.5	
TriGly (mmol/l)	0.8 ± 0.2	1.2 ± 0.5	1.2 ± 1.0	
Adiponectin (ng/ml)	7432 ± 3606	9382 ± 5273	10267 ± 5350	
Leptin (pg/ml)	23225 ± 10977	18571 ± 13705	12483 ± 8672	
Resistin (ng/ml)	9.4 ± 2.0	10.2 ± 1.2	9.7 ± 1.4	
Ins (mmol/l)	5.3 ± 3.3	8.0 ± 4.5	6.2 ± 3.6	
Gluc (mmol/l, plasma)	4.2 ± 0.4	5.2 ± 0.7	5.0 ± 0.7	a, b
E2 (pmol/l)	496.5 ± 311.9	34.8 ± 28.4	183.0 ± 211.3	a^#^, b^#^, c^#^
E1 (pmol/l)	369.4 ± 195.3	100.9 ± 26.0	962.0 ± 1517.8	a^#^, c^#^
T (nmol/l)	1.02 ± 0.30	0.66 ± 0.27	0.74 ± 0.31	a, c
SHGB (nmol/l)	49.3 ± 17.6	44.1 ± 11.6	72.3 ± 31.1	c
LH (IUl)	12.8 ± 13.2	39.9 ± 30.2	31.6 ± 17.3	a^#^, b^#^
FSH (IUl)	6.22 ± 2.85	83.7 ± 32.7	57.0 ± 31.2	a, b, c

Significant comparisons between the studied groups are marked with letters a) Pre vs post No HRT b) Pre vs post HRT c) Post HRT vs post No HRT. Independent samples T-test was used for a and b comparisons and paired samples T-test for c comparisons when variables were parametric. ^#^Mann Whitney U test (a and b) and Wilcoxon matched pair signed-rank test (c) was used for non-parametric variables. P < 0.05 was considered significant. Results are shown as mean ± S.D. BMI: body mass index, hsCRP: high sensitive c-reactive protein, IL-10: interleukin 10, IL-6: interleukin 6, TNFa: tumor necrosis factor alpha WBC: white blood cell count, RBC: red blood cell count, HGB: hemoglobin, Plt: platelet, Chol: cholesterol, LDL: low density lipoprotein, HDL: high density lipoprotein, TriGly: triglycerides, E2: 17β-estradiol, E1: estrone, T: testosterone, SHGB: sex hormone-binding globulin, LH: lutenizing hormone, FSH: follicle stimulating hormone.

**Table 2:** Read counts of the differentially expressed miRs based on sequencing results.

miR	Pre	Mean count		Overdispersion	Comparison (p-value, FDR)		
		No HRT	HRT		No HRT vs. Pre	HRT vs. Pre	HRT vs. No HRT
miR-126-5p	3942	5822	5601	0.002	<0.001**↑**	<0.001**↑**	<0.001**↓**
miR-142-5p	10591	6254	6669	0.007	<0.001**↓**	<0.001**↓**	<0.001**↑**
miR-484	2106	1201	1471	0.029	<0.001**↓**	<0.001**↓**	<0.001**↑**
miR-10b-5p	8535	6958	5323	0.007	<0.001**↓**	<0.001**↓**	<0.001**↓**
miR-532-5p	395	263	356	0.102	0.184	1	<0.001**↑**
miR-1285-3p	72	40	97	1.28	1	1	<0.001**↑**
miR-30a-5p	1121	1880	1485	0.101	0.065	1	0.001**↓**
miR-3688-3p	6	4	10	6.248	1	1	0.016**↑**
miR-29b-3p	0	6	17	6.436	0.934	0.494	0.029**↑**
miR-106b-5p	399	219	333	0.155	0.118	1	0.029**↑**
miR-29c-3p	174	154	209	0.201	1	1	0.033**↑**
miR-1306-5p	4	2	5	4.692	1	1	0.036 **↑**
miR-148a-3p	2095	3863	2814	0.094	0.008 **↑**	1	0.049**↓**
miR-301a-3p	12	38	22	2.061	1	1	0.049**↓**
miR-375	1056	629	329	0.482	1	0.045**↓**	0.115
miR-28-3p	319	607	443	0.103	0.009**↑**	1	0.275
miR-27b-3p	3307	8419	7025	0.034	<0.001**↑**	<0.001**↑**	0.325
miR-10a-5p	3958	7682	6252	0.029	<0.001**↑**	<0.001**↑**	0.671
miR-215-5p	190	1010	592	0.151	<0.001**↑**	<0.001**↑**	0.671
miR-144-5p	585	375	433	0.034	<0.001**↓**	<0.001**↓**	1
miR-186-5p	2056	2362	2598	0.026	0.063	<0.001**↑**	1

The average read counts of the studied groups are presented on the left (Pre: premenopausal women, HRT: postmenopausal hormone replacement therapy users, No HRT: postmenopausal non-users). FDR corrected group comparisons are presented on the right. Arrow head pointing up indicates upregulation and arrow head pointing down downregulation in the first group of the comparison. Overdispersion is a measure of a greater variability.
